# Creation of the bone bank of the Rabat and Casablanca region in Morocco

**DOI:** 10.11604/pamj.2018.29.210.14343

**Published:** 2018-04-11

**Authors:** Mohammed Chahbouni, Amal Rami, Mohammed El Morhit, Mohammed Kharmaz, Mohammed salah Berrada, Moradh El Yaacoubi

**Affiliations:** 1 Faculty of Medicine and Pharmacy, Mohammed-V University, Rabat, Morocco

**Keywords:** Bone allograft, bone bank, bone reconstruction

## Abstract

For a long time the use of bone grafting has demonstrated its interest in orthopedic surgery and traumatology. The autografts which are still very frequently used present various problems. On the one hand, it is necessary to find a correct mechanical quality and a sufficient quantity of bone. On the other hand, the graft removal lengthens the operative time and generally painful in postoperative. These disadvantages of autografts have led to the development of bone allografts. Indeed, the low immunogenic power of the bone, the good integration of the graft and the ease of bone preservation techniques make it possible to overcome the various problems posed by bone autografts. The increasing use of bone allografts has resulted in the need for a structure allowing the management of graft stocks. The purpose of this work is to demonstrate the mode of operation of a bone bank, whose conservation activity is limited to the femoral heads treated by cryopreservation and without secondary sterilization process. The bank collaborates with all orthopedic surgeons in the Rabat and Casablanca city at first and then with all orthopedic surgeons in Morocco. It provides allografts in quality and safety.

## Perspective

The use of bone allografts has emerged in the face of the growing need for bone grafts in orthopedic surgery, traumatology and dental surgery. This need is not met by autografts which, despite their good results in terms of integration, pose the problem of comorbidity. Xenografts are avoided due to the risk of transmission of spongiform infection, while the remaining bone substitutes, which remain the most available, still pose the problem of toxicity and biocompatibility [1, 2]. Biologically, allografts are considered to be excellent transplant material as immune reactions have only a secondary clinical role, with rare cases of rejection. The bone bank is defined as a service of a State-accredited public health institution that takes care of the collection, collection, securing, distribution and traceability of bone allografts. Bone allografts are of two types: femoral heads taken from a living donor during hip arthroplasty, or massive bone segments from deceased donors [3].

**Legal and ethical aspects:** The Moroccan law on the donation, taking and transplantation of human organs and tissues only appeared in 1999 and it's implementing decrees in 2003. The donation must be made in compliance with the most important rules namely anonymity, gratuity and voluntary service. Femoral heads taken during arthroplasty are considered as operative residues, the management of which is strictly reserved for tissue banks belonging to public hospital structures approved by the Ministry of Health. Law 16-98 did not treat the case of operative residues separately, including them in the texts of the human tissues that regenerate and consequently the private clinics which will cooperate with the Rabat bone bank must be approved to realize these samples and transplant femoral heads. This approval is granted by the Ministry of Health on the proposal of the National Council of Physicians [1-4]. From a religious view, Islam encourages and authorizes organ donation and regards it as a gesture of benevolence and solidarity. It also makes it possible to profit from an organ amputated from the body because of illness provided that it does not make it into a commercial transaction object.

**Figure 1:**
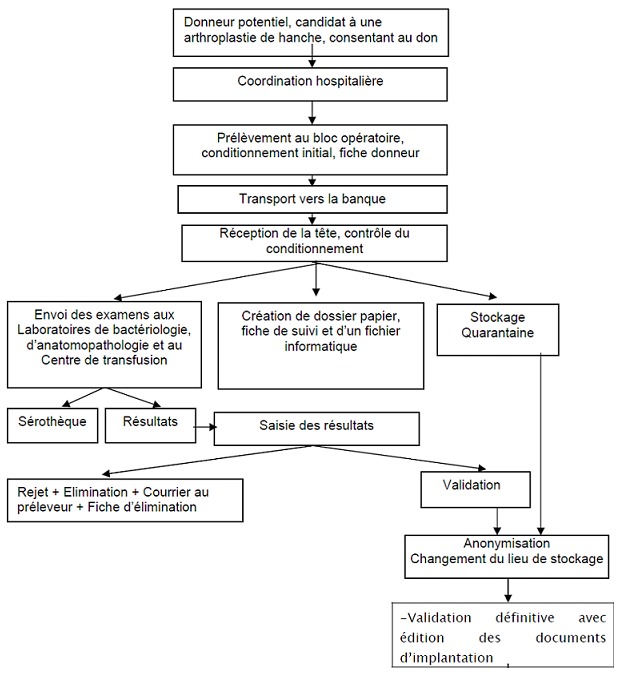
Internal circuit of the bone bank

**Figure 2:**
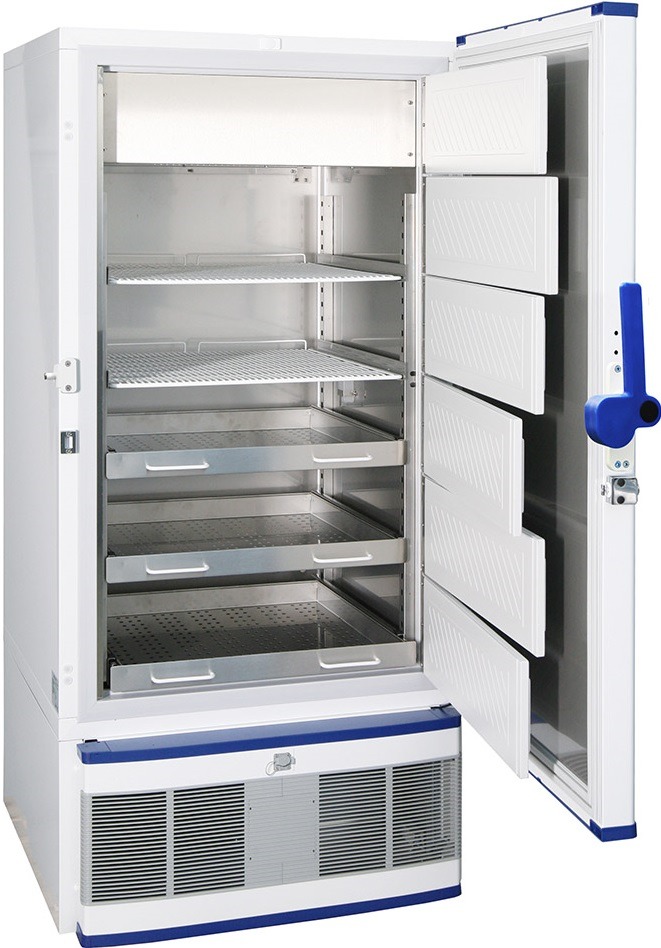
Freezers for femoral heads

**Organizational and technical aspects:** Our project consists in realizing a bone bank whose conservation activity is restricted for technical and legal reasons to the femoral heads taken from a living donor and treated by cryopreservation without a secondary sterilization process, knowing that the femoral heads presently constitute in our context, the allograft that is the easiest to obtain, since the samples taken from the deceased human are not entirely accepted by public opinion. The femoral heads have the status of operative residues, which facilitates the obtaining of the donor's consent; they are taken during the hip replacements which are carried out more and more in all the hospitals of Morocco which guarantees a sufficient supply for the bone bank. Being given that prosthetic surgery is a programmed surgery this allows a good selection of donors. The staff of the bank must be qualified and in sufficient number to follow up the procedures and control activities required for a quality service. The staff will consist of a bank manager, a medical doctor and his deputy, a biologist or a pharmacist, three nurses and a medical secretary. Only the bank manager and his deputy are authorized to validate the femoral heads. The necessary equipment includes: three freezers allowing freezing at less 80^°^C with a temperature safety system, two freezers are needed to isolate the validated femoral heads from the quarantined femoral heads (Figure 1). Sterile pots for conditioning the femoral heads with isothermal containers for transport of the grafts at a temperature between -4 and +4 degree (Figure 2). The implantation of the bone bank is at the Ibn Sina University Hospital Center in Rabat, with a room for storing grafts with a seroscope, a secretariat and a paper storage room. The bone bank operates as a non-profit cooperative, which surgeons contribute to feeding through femoral heads taken from arthroplasties. The bone bank in return provides them with quality secure transplants. The role of the bank begins with hospital coordination with the sampling team [5]. The packaging is done at the place of collection by placing the femoral head in sterile pots provided by the bank, the labelling of which contains all the information necessary for the traceability of the graft and the mention of tissue of human origin for therapeutic use. Transport must be carried out in containers that maintain the temperature between -4^°^C and + 4^°^C, it is usually carried out by a specialized transport company, but if the transport is immediate, it may be carried out by an ambulance driver, as is the case with blood products. During the reception of the head at the bank, the staff check the packaging, put the head in the specific quarantine freezer, send the samples to the laboratories of bacteriology and anatomopathology of the Ibn Sina University Hospital Center and the blood transfusion centre of the Rabat, keeps a serum sample serum and creates a paper file with a tracking sheet and a computer file [6-8].

**Figure 3:**
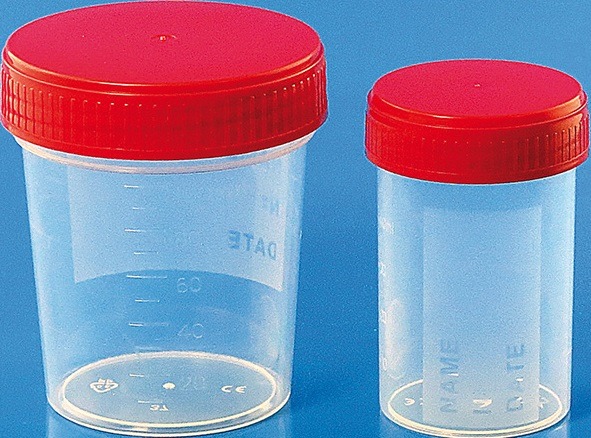
Sterile pots

The choice of cryopreservation comes from financial as well as technical requirements; cryopreservation is cheaper than freeze-drying, but it is also a more feasible process for staff, with better bone quality. Similarly, the choice of the “no touch” option of not performing secondary sterilization is due to the fact that irradiation, which remains the best sterilization process, impairs the mechanical properties of the graft while the cryopreserved femoral heads, without secondary sterilization have demonstrated their reliability in terms of health safety, provided that they comply with the aseptic measures during sampling, perform a selection of donors and respect the quarantine period [6-9]. The safety of the grafts thus passes through an anamnestic, clinical and biological screening. Biological screening involves bacteriological examination and pathological examination on small fragments of the femoral head, as well as a series of serological tests with seroconversion control six months later. There are four serologic serological tests: HIV serology, hepatitis B and C serology and syphilitic serology. If the recipient patient is immunosuppressed, a complementary cytomegalovirus (CMV) and Epstein bar virus (EBV) serology is required (based upon serum sample stored in the Serum Bank). The quarantine imposed by the law can only be lifted if, in addition to the negativity of the clinical exclusion criteria and the negativity of the initial assessment, the serological control of six months is negative [8-10]. On returning from the examinations six months after the end of the quarantine period, the heads are either rejected if the serology returns positive or validated otherwise, with anonymization, change of the place of storage, implementation, transport and traceability [11]. The head is dispatched at the request of the implanting surgeon who must return after the transplant a recipient card to the bank for traceability, which corresponds to all the information and measures taken to quickly track and trace all the steps from the clinical examination of the donor to the therapeutic use of the graft. Its implementation is a legal obligation [11] (Figure 3).

**Interest of the project in the region:** The bank of cryopreserved femoral heads initially aims at supplying public and private healthcare facilities in Rabat and Casablanca regions. Subsequently, as soon as the stocks and the implementation of the procedures allow, the bank will be able to supply the entire Moroccan kingdom. In order to judge the feasibility of the project, an approach of orthopedic surgeons was established. Our survey was carried out among 100 orthopedic surgeons throughout the kingdom, 80 of them from the Rabat and Casablanca region. The opinion survey concluded that the project was beneficial, with a need for allografting in trauma, then in tumor surgery and in 3^rd^ place during revision of arthroplasty. The orthopedic surgeons contacted said they were ready to collaborate with the Rabat bank and to supply it. The approach of patient's candidates for hip arthroplasty has also been carried out to judge the patients' adhesion to the donation of their femoral head. It should be remembered that the sampling, even if it is a surgical residue, does not can be achieved without the informed consent of the patient. We surveyed 60 patients who were candidates for hip arthroplasty: 90% of the patients said they were in favour of the donation and were ready to perform the serological tests.

## Conclusion

The bone bank project is part of a therapeutic need due to the growing need for bone allografts in trauma, oncology and arthroplasty. The choice of a femoral head bank is currently the most accessible in our context, both financially and legally. The femoral heads will allow orthopedic surgeons to improve their practice and expand their therapeutic arsenal by offering more conservative treatments and limiting the flow of patients abroad for costly care.

## Competing interests

The authors declare no competing interest.
